# Impact of Viruses on Prokaryotic Communities and Greenhouse Gas Emissions in Agricultural Soils

**DOI:** 10.1002/advs.202407223

**Published:** 2024-10-07

**Authors:** Xing Huang, Lucas P. P. Braga, Chenxiao Ding, Bokai Yang, Tida Ge, Hongjie Di, Yan He, Jianming Xu, Laurent Philippot, Yong Li

**Affiliations:** ^1^ Zhejiang Provincial Key Laboratory of Agricultural Resources and Environment College of Environmental and Resource Sciences Zhejiang University Hangzhou 310058 China; ^2^ Department of Plant Sciences University of Cambridge Cambridge CB2 3EA UK; ^3^ State Key Laboratory for Managing Biotic and Chemical Threats to the Quality and Safety of Agro‐products Institute of Plant Virology Ningbo University Ningbo 315211 China; ^4^ Université Bourgogne INRAE Institut Agro Dijon Agroécologie Dijon 21000 France

**Keywords:** agricultural soil, biogeochemical cycles, greenhouse gas emission, microbial functional trait, soil viruses, viral shunt

## Abstract

Viruses are abundant and ubiquitous in soil, but their importance in modulating greenhouse gas (GHG) emissions in terrestrial ecosystems remains largely unknown. Here, various loads of viral communities are introduced into paddy soils with different fertilization histories via a reciprocal transplant approach to study the role of viruses in regulating greenhouse gas emissions and prokaryotic communities. The results showed that the addition of viruses has a strong impact on methane (CH_4_) and nitrous oxide (N_2_O) emissions and, to a minor extent, carbon dioxide (CO_2_) emissions, along with dissolved carbon and nitrogen pools, depending on soil fertilization history. The addition of a high viral load resulted in a decrease in microbial biomass carbon (MBC) by 31.4%, with changes in the relative abundance of 16.6% of dominant amplicon sequence variants (ASVs) in comparison to control treatments. More specifically, large effects of viral pressure are observed on some specific microbial communities with decreased relative abundance of prokaryotes that dissimilate sulfur compounds and increased relative abundance of *Nanoarchaea*. Structural equation modeling further highlighted the differential direct and indirect effects of viruses on CO_2_, N_2_O, and CH_4_ emissions. These findings underpin the understanding of the complex microbe‐virus interactions and advance current knowledge on soil virus ecology.

## Graphical abstract

Viruses in soil significantly influence greenhouse gas emissions, yet their role remains understudied. Varying viral loads are introduced into paddy soils with different fertilization histories to investigate their impact. The results show that viruses strongly affect methane and nitrous oxide emissions, microbial communities, and dissolved carbon and nitrogen pools, highlighting the complex interactions between soil viruses and ecosystem processes.

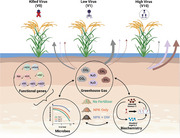

## 1 Introduction

Viruses are extremely diverse and the most abundant biological entities on earth, with an estimated 10^30^ virus‐like particles globally.^[^ ^1^ ^]^ They are highly host‐specific with a very narrow range of microbial hosts.^[^ ^2^ ^]^ Several studies have documented the significant influence of viruses on their host communities in aquatic ecosystems.^[^ ^1^, ^3^ ^]^ For example, in the oceans, viral infections contribute to 20–40% of bacterial mortality daily and cause the release of 150 gigatons of carbon annually.^[^ ^1a^ ^]^ Viruses can also infect archaea and manipulate host energy metabolism, as shown by the recent identification of soil viruses propagating in archaeal ammonia oxidizers.^[^ ^4^ ^]^ However, although soil contain up to 10^10^ virus‐like particles per gram of soil, knowledge of the role of viruses in terrestrial ecosystems is lagging, partly due to methodological challenges related to the heterogeneity of the soil matrix.^[^ ^5^ ^]^

The influence of viruses on their host communities is manifold. Viral lysis can affect prokaryotic community diversity and composition via top‐down controls.^[^ ^6^ ^]^ The “Kill the Winner (KtW)” hypothesis suggests that viruses could even have a central role in modulating bacterial diversity by predominantly preying on fast‐growing bacteria.^[^ ^7^ ^]^ Studies coupling DNA stable‐isotope probing and metagenomic analyses have provided evidence of viral infections in soil prokaryotes that play key roles in methane oxidation and nitrification.^[^ ^4^, ^8^ ^]^ Viruses can also contribute to biogeochemical cycling by releasing organic matter and nutrients during the lysis of host cells, a process known as the viral shunt.^[^ ^5^, ^9^ ^]^ Moreover, it has been shown that viral‐encoded auxiliary metabolic genes (AMGs) not only modulate host metabolism to facilitate virus replication efficiently but also potentially affect nutrient cycling.^[^ ^10^ ^]^ Several viral‐encoded AMGs linked to carbon and nitrogen cycles have been identified in the last decade, and a recent study demonstrated that these AMGs produce functional, active proteins.^[^ ^10^, ^11^ ^]^ However, direct demonstrations of alterations in biogeochemical cycling due to soil viruses are still scarce.^[^ ^12^ ^]^

Land use and farming practices can have a strong impact on terrestrial ecosystems.^[^ ^13^ ^]^ Thus, numerous studies have reported changes in soil microbial diversity, biogeochemical cycling, and greenhouse gas emissions in response to fertilization.^[^ ^14^ ^]^ For example, the addition of nitrogen fertilizers in agriculture significantly influences emissions of methane (CH_4_) and nitrous oxide (N_2_O), two major greenhouse gases.^[^ ^15^ ^]^ Paddy fields cover less than 10% of the global cropland area yet are a major source of GHG emissions, contributing up to 19% and 11% of the total anthropogenic emissions of CH_4_ and N_2_O, respectively.^[^ ^16^ ^]^ Recent studies have shown that land use changes can also shape soil viral communities, with bacterial communities, pH, moisture, and nutrients being the major determinants.^[^ ^13^, ^15^ ^]^ While a considerable number of studies has shown that changes in soil pH, nutrient pools, and microbial communities due to nitrogen fertilizer application have differential effects on GHG emissions, the extent to which these anthropogenic GHG emissions are modulated by viral infections remains unknown.^[^ ^15^, ^16^, ^17^ ^]^

**Figure 1:**
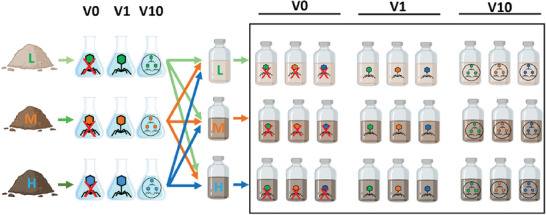
Schematic illustration of the experimental design. Viral fractions from soil suspensions were obtained and concentrated using a tangential flow filtration (TFF) system (see Experimental Section). Two different concentrations were considered (V1 and V10) and compared against the control (V0) obtained by autoclaving the viral suspension. Viral suspensions were inoculated in native and non‐native soils in a transplant design. Three soils with different fertilization histories were used (L: without fertilizers; M: with mineral fertilizers; H: with mineral and organic fertilizers), making a total of 27 treatments with four replicates. Viruses are represented in green, orange, and blue according to their soil sources. A red cross indicates the control prepared with autoclaved V10.

Here, we aim to assess the impact of viruses on prokaryotic communities, biogeochemical cycles, and greenhouse gas emissions in paddy soils with different histories of long‐term fertilization. For this purpose, we manipulated soil virus communities by introducing viruses extracted from different soils as well as inactivated viruses (noted as V0) into soil microcosms, using a reciprocal transplant experiment (**Figure** **1**). As viral abundance in soils can vary by several orders of magnitude, two different concentrations of viruses were added to the soils (V1 and V10).^[^ ^18^ ^]^ We focused on carbon and nitrogen cycling and combined measurements of soil nutrients as well as carbon dioxide (CO_2_), CH_4_, and N_2_O emissions along with the quantification of the functional genes involved in C and N cycling and the sequencing of the prokaryotic community. Specifically, we sought 1) to characterize the impact of the addition of different amounts of native and non‐native viruses on the diversity and composition of prokaryotic communities in paddy soils with different histories of fertilization regimes, 2) to understand how the response of soil microbes to virus loads affects nutrients cycling as well as GHG emissions, and 3) to identify the main drivers of shifts in GHG emissions following virus addition.

## 2 Results

### 2.1 Characterization of the Added Viral Solutions

The images of transmission electron microscopy (TEM) and epifluorescence‐microscopy (EFM) showed that with a series of filtration and dilution sterilization steps, we obtained viral solutions with diverse morphologies and varying concentrations (Figures S1 and S2, Supporting Information). The amended virus suspensions were dominated by *Caudoviricetes* (85.5%) regardless of the soil history (Figure S3, Supporting Information). However, significant differences in the viral community structure were observed between the virus suspensions obtained from the three types of soil (*p* < 0.001, PERMANOVA, Figure S3, Supporting Information).

### 2.2 Impact of Viruses on Soil Nutrient Content

**Figure 2:**
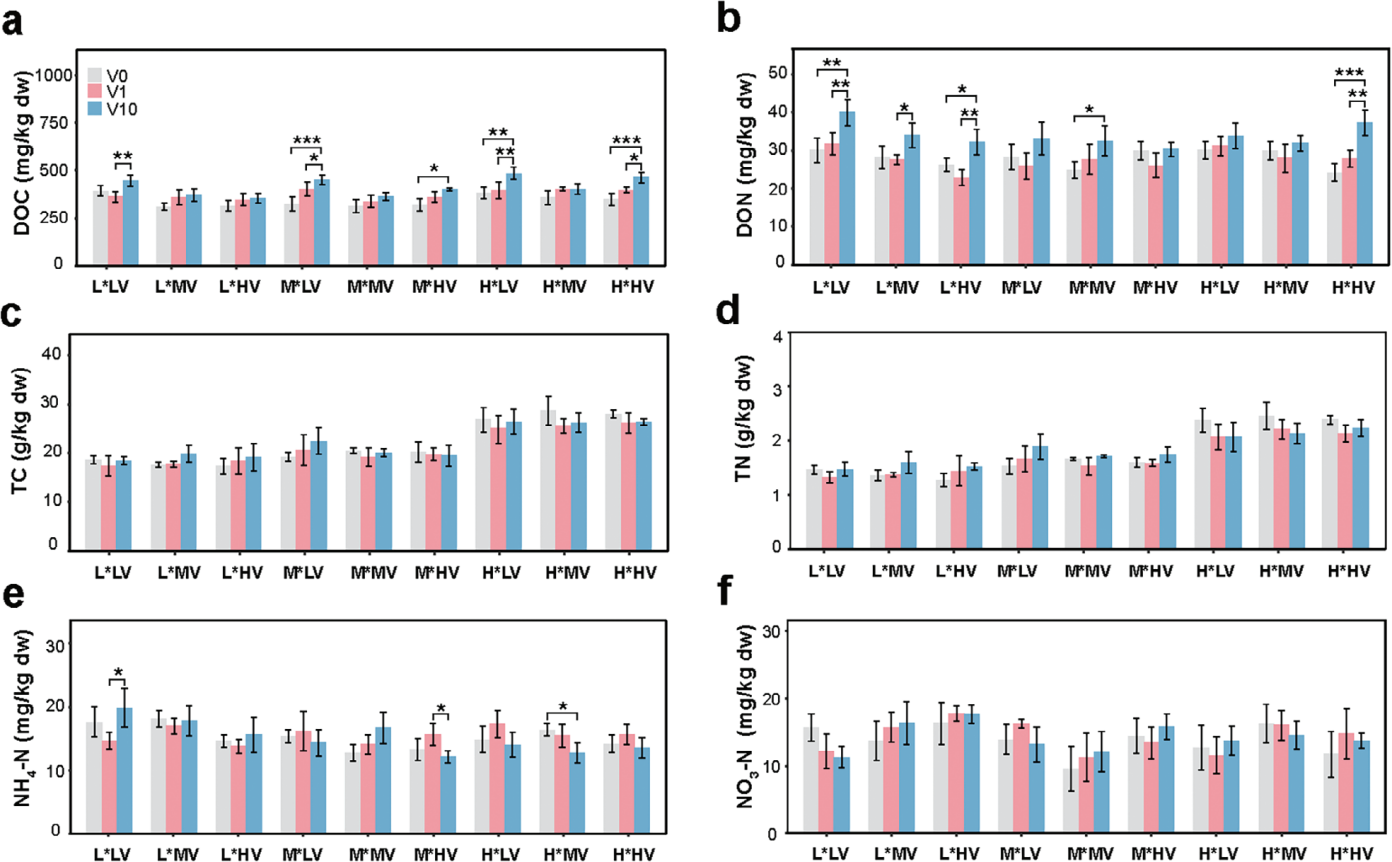
Soil carbon and nitrogen content after viral suspension inoculation. The plots represent the concentrations of a) dissolved organic carbon (DOC), b) dissolved organic nitrogen (DON), c) total carbon (TC), d) total nitrogen (TN), e) ammonium (NH_4_^+^‐N), and f) nitrate (NO_3_^−^‐N) after the 8‐week incubation period. The *y*‐axis shows nutrient contents, and the x‐axis indicates the different treatments labeled according to the soil (L|M|H) followed by the source of the virus in the suspension that it was inoculated with (LV|MV|HV); e.g., treatment L^*^LV indicates soil L inoculated with virus suspension from soil L (LV). The green, orange, and blue bars indicate the viral loads of V0, V1, and V10, respectively. The asterisk inside the plots indicates significant differences (Tukey's HSD test, ^*^ *p* < 0.05; ^**^ *p* < 0.01; ^***^ *p* < 0.001).

After 8 weeks of incubation, we observed differential effects of virus addition on soil nutrient content depending on the viral load, virus source, and fertilization history (**Figure** **2** and Table S2, Supporting Information). A three‐way ANOVA revealed that the viral load significantly influenced dissolved organic carbon (DOC) and nitrogen (DON) content as well as the ammonium (NH_4_^+^‐N) concentration in the soil (*p *< 0.05). Notably, the highest viral load resulted in increased concentrations of DOC and DON in at least half of the treatments (Figure 2). The concentration of DON was also significantly affected (*p *< 0.001) by the virus source (i.e., the addition of viruses from native or nonnative soils), with increased DON in the fertilized soils only after the addition of a high load of native viruses (Figure 2). Additionally, both DOC and DON were significantly affected by the interaction between the virus source and soil fertilization history (*p *< 0.001). In contrast, neither the viral load nor the virus source affected the total carbon (TC), total nitrogen (TN), and nitrate (NO_3_^−^‐N) concentrations (*p* > 0.05).

### 2.3 Impact of Viruses on Greenhouse Gas Emissions

**Figure 3:**
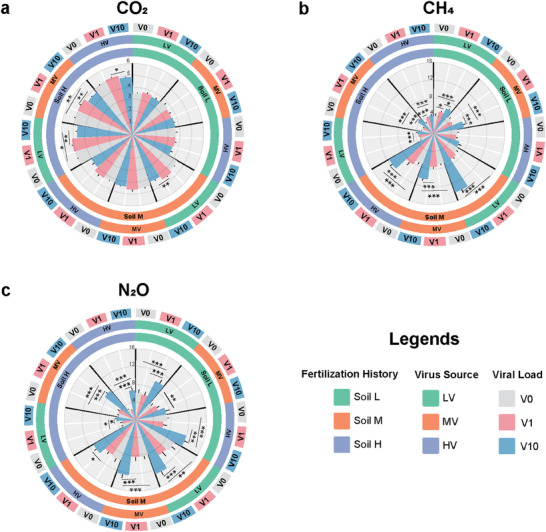
Soil greenhouse gas emissions after viral suspension inoculation. The outer, middle, and inner circles indicate the viral load (V0, V1, and V10), virus source (LV, MV, and HV), and soil fertilization history (L, M, and H), respectively. The y‐axis shows cumulative emissions in milligrams per kilogram of dry‐weight soil. a) CO_2_ (*Y*‐axis^*^1000 mg k^−1^g^−1^ dw); b) CH_4_ (*Y*‐axis mg k^−1^g^−1^ dw); c) N_2_O (*Y*‐axis mg k^−1^g^−1^ dw). The asterisk on the plots indicates significant differences. (Tukey's HSD test, ^*^ *p* < 0.05; ^**^ *p* < 0.01; ^***^ *p* < 0.001).

In general, the emission rates of greenhouse gases (GHGs) displayed temporal variations, with peak emissions occurring during the initial 7 days for CO_2_ (Figure S4, Supporting Information) and CH_4_ (Figure S5, Supporting Information) and from Days 10 to 35 for N_2_O (Figure S6, Supporting Information). As expected, soil fertilization history emerged as a significant driver of GHG emissions (**Figure** **3** and Table S3, Supporting Information). The highest cumulative CO_2_ emissions were observed in soil H (fertilized with 70% NPK + 30% chicken manure) compared to unfertilized soil L and soil M (fertilized with NPK only) (*p* < 0.001, ANOVA). (Figure 3a and Table S3, Supporting Information). In contrast, significantly lower methane and nitrous oxide emissions were observed in soil H than in the other two soils.

Viral load had a significant impact on the emissions of all three greenhouse gases (*p* < 0.001, ANOVA, Table S3, Supporting Information). Specifically, the addition of a high load of virus (V10) increased N_2_O emissions in all treatments by 13.5–187.1%, while CH_4_ emissions increased between 13.9–121.3%, except in the treatment corresponding to the addition of the virus from soil H to soil L (Figure 3b,c). In contrast, we found a weaker effect on CO_2_ emissions, with a slight increase in CO_2_ emissions with the V1 addition and an obvious decrease with the V10 addition (Figure 3a and Table S3, Supporting Information), while the inhibition of soil respiration by V10 on V1 was observed only in soil H. No significant effect of virus source on GHG emissions was observed, and the interaction between virus source and viral load or soil fertilization history had a significant influence only on CH_4_ emissions (Table S3, Supporting Information).

### 2.4 Impact of Viruses on Functional Genes Associated with Carbon and Nitrogen Cycling

**Figure 4:**
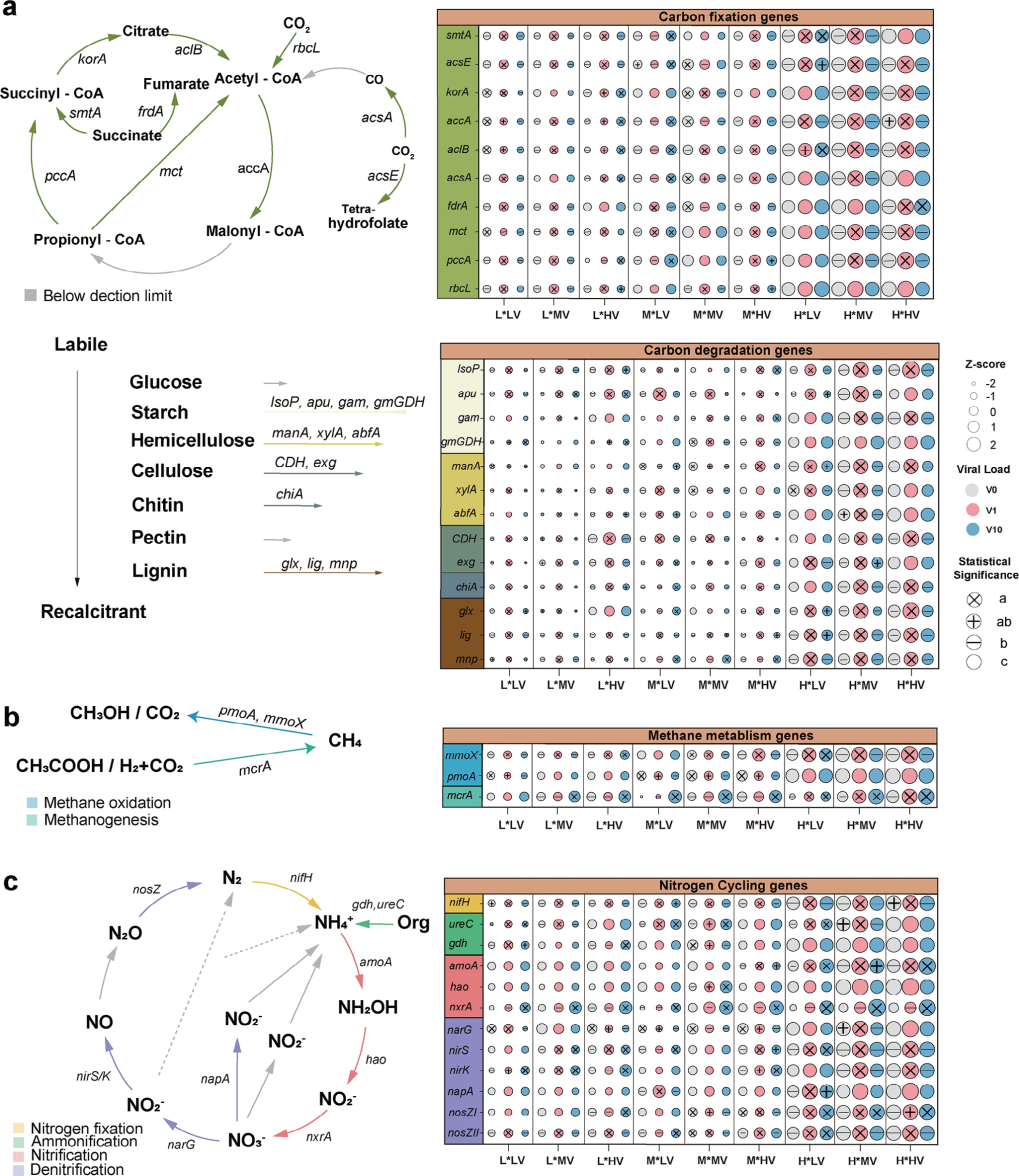
Responses of genes involved in soil biogeochemical cycling to inoculation with viral suspensions. Quantification by QMEC of the genes involved in a) carbon fixation and degradation, b) methane metabolism, and c) nitrogen metabolism. The metabolic pathways are represented on the left. The size of the circles in the right panel represents the normalized abundance of functional genes, and their colors represent the viral load (V0, V1, and V10). Different symbols inside the circles denote significant differences (Tukey's HSD test *p* < 0.05).

A total of 26 functional genes related to carbon cycling were detected via the high‐throughput quantitative‐PCR‐based chip (QMEC) assay, including 10 genes associated with carbon fixation, 13 genes related to carbon degradation, and 3 genes involved in methane metabolism (**Figure** **4**). The abundance of most of these functional genes was primarily influenced by the soil fertilization history, but significant changes in the abundance of carbon fixation and carbon degradation genes were also observed in response to the viral load (Tables S4 and S5, Supporting Information). Notably, the abundance of most genes was higher in the low viral load treatments (V1) than in the control (V0) and high viral load treatments (V10), mirroring the trend observed for the cumulative CO_2_ emissions (Figure 4a). Analysis of genes encoding enzymes important for CH_4_ emissions revealed contrasting changes in the abundances of methanogens (*mcrA*) and methanotrophs (*pmoA* and *mmoX*) induced by the viral load (Figure 4b and Table S6, Supporting Information). Specifically, the addition of a higher viral load significantly increased the abundance of *mcrA* in all soils while decreasing the abundance of *pmoA* in soil M and soil L after the addition of native viruses. In contrast, the *mmoX* gene encoding the soluble form of methane monooxygenase was more predominant in soils inoculated with a low viral load compared to the control (V0) and those inoculated with a higher viral load (V10) (Figure 4b).

We also monitored functional genes involved in four key nitrogen cycling processes, i.e., nitrogen fixation (*nifH*), ammonification (*ureC* and *gdh*), nitrification (*amoA*, *hao*, and *nxrA*), and denitrification (*narG*, *napA, nirS*, *nirK*, *nosZI*, and *nosZII*). As expected, soil fertilization history had a significant impact on all measured N‐cycling genes, with higher abundances in soil H (*p *< 0.001, Figure 4c and Table S7, Supporting Information). Moreover, we found that viral load also had a small but significant effect on the abundance of all targeted nitrogen cycling genes, except for *gdh* and *hao* (Figure 4c and Table S7, Supporting Information). In particular, the strongest response was observed in nitrite oxidizers after the addition of a high viral load in all treatments, with an increase in *nxrA* of 1.41 to 3.91 folds (Figure 4c). In contrast, the virus source only had a significant effect on the abundance of the *nifH, amoA*, and *narG* genes (*p *< 0.05, Table S7, Supporting Information).

### 2.5 Responses of the Microbial Community to Virus Addition

**Figure 5:**
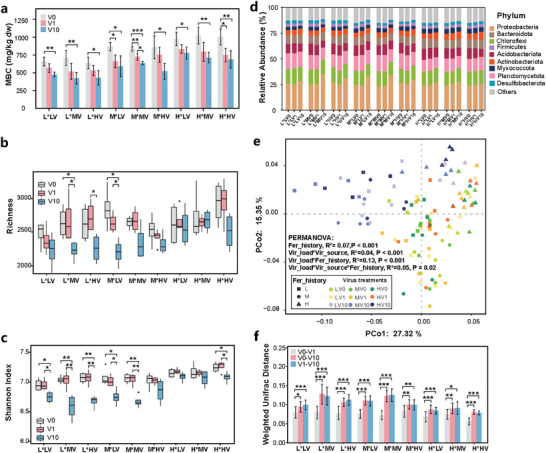
Shifts in soil microbial communities after inoculation with viral suspensions. a) Soil microbial biomass carbon (MBC), b) richness, and c) Shannon index of prokaryotic communities at the ASV level in soils. The x‐axis in a), b), and c) represent treatment labels; e.g., L^*^LV indicates the virus suspension obtained from soil L (LV) inoculated into soil L. d) Changes in the relative abundance of prokaryotic communities at the phylum level in soils. The x‐axis represents the treatment label, including the viral load that they received. e) Principal coordinate analysis (PCoA) based on weighted UniFrac distance illustrates the variation in microbial community diversity across treatments. Permutational multivariate analysis of variance (PERMANOVA) was performed to test individual and interactive effects (represented by “^*^”) of viral load, virus source, and soil fertilization history on community structure. f) Weighted UniFrac distance values across treatments. An asterisk on a bar or box indicates a significant difference (Tukey's test, ^*^ *p* < 0.05; ^**^ *p* < 0.01; ^***^ *p* < 0.001).

We found that the soil MBC content decreased by 19.8–40.3% with increasing viral load for all three soils (**Figure** **5**a). 16S rRNA gene profiling yielded the identification of a total of 8706 prokaryotic amplicon sequence variants (ASVs), with *Proteobacteria*, *Chloroflexi*, *Firmicutes*, *Acidobacteria*, and *Actinobacteria* representing the most dominant phyla (Figure 5d). We observed an increase in the relative abundance of *Proteobacteria* under high viral load compared to low and no viral load treatments in soil L and M, but not in soil H. The analysis of α‐diversity showed that the addition of a high viral load (V10) significantly decreased both the richness and Shannon index in soils L and M, and those in soil H only with native virus addition (H^*^HV) (Figure 5b,c and Table S8, Supporting Information). Shifts in prokaryotic community structure were consistent with bacterial diversity patterns, as significant differences were mostly observed between high viral load and low viral load or the inactivated virus control (Figure 5e,f). The interaction between soil fertilization history and viral load explained the greatest variation in community composition (*R* ^2^ = 0.13, *p *< 0.001, PERMANOVA).

**Figure 6:**
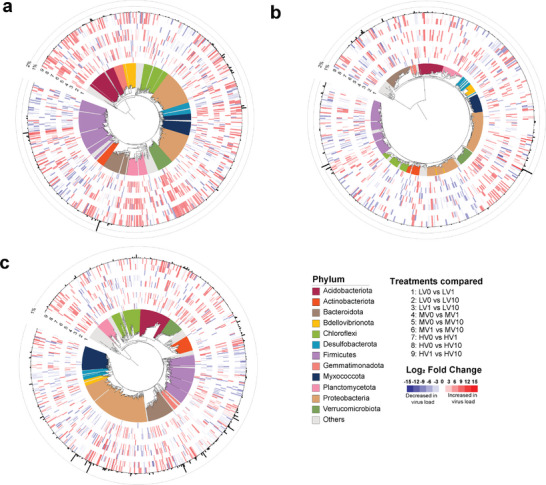
Taxonomic assignment and distribution of significantly impacted prokaryotic ASVs by virus addition in a) soil L, b) soil M, and c) soil H. Heatmaps showed the log2‐fold changes in the relative abundances of significantly increasing (red shades) and decreasing (blue shades) ASVs for each of the 9‐group comparison. The ASV taxonomy is indicated by different colors on the innermost ring. Bars in the outer circle depict the relative abundance of each ASV in the soil at the time of sampling (see Tables S9–S11, Supporting Information).

To further identify the ASVs that were affected by virus addition, we conducted a differential abundance analysis using DESeq2 to estimate significant differences in the relative abundance of dominant ASVs between treatments (**Figure** **6**). The number of ASVs affected by virus addition was larger at high viral loads than at low viral loads (Figure 6 and Table S9, Supporting Information). Thus, we found that on average, 0.7% and 16.2% of the dominant ASVs were affected by low and high viral loads, respectively. Our analysis revealed that these differentially affected ASVs cover 36 phyla, mostly *Proteobacteria* and *Firmicutes* (Tables S10–S12, Supporting Information). Interestingly, all 30 ASVs belonging to *Nanoarchaeota* exhibited increased relative abundances after virus addition. In contrast, 90.4% of the ASVs belonging to the *Desulfarculaceae*, *Desulfocapsaceae*, and *Syntrophobacteraceae* families within the phylum *Desulfobacterota*, as well as to the *Desulfotomaculales* and the *Desulfitobacteriacea* families within the phylum *Firmicutes*, were negatively affected at high viral load (Figure 6 and Tables S10–S12, Supporting Information).

### 2.6 Drivers of the Effect of Virus Addition on GHG Emissions

**Figure 7:**
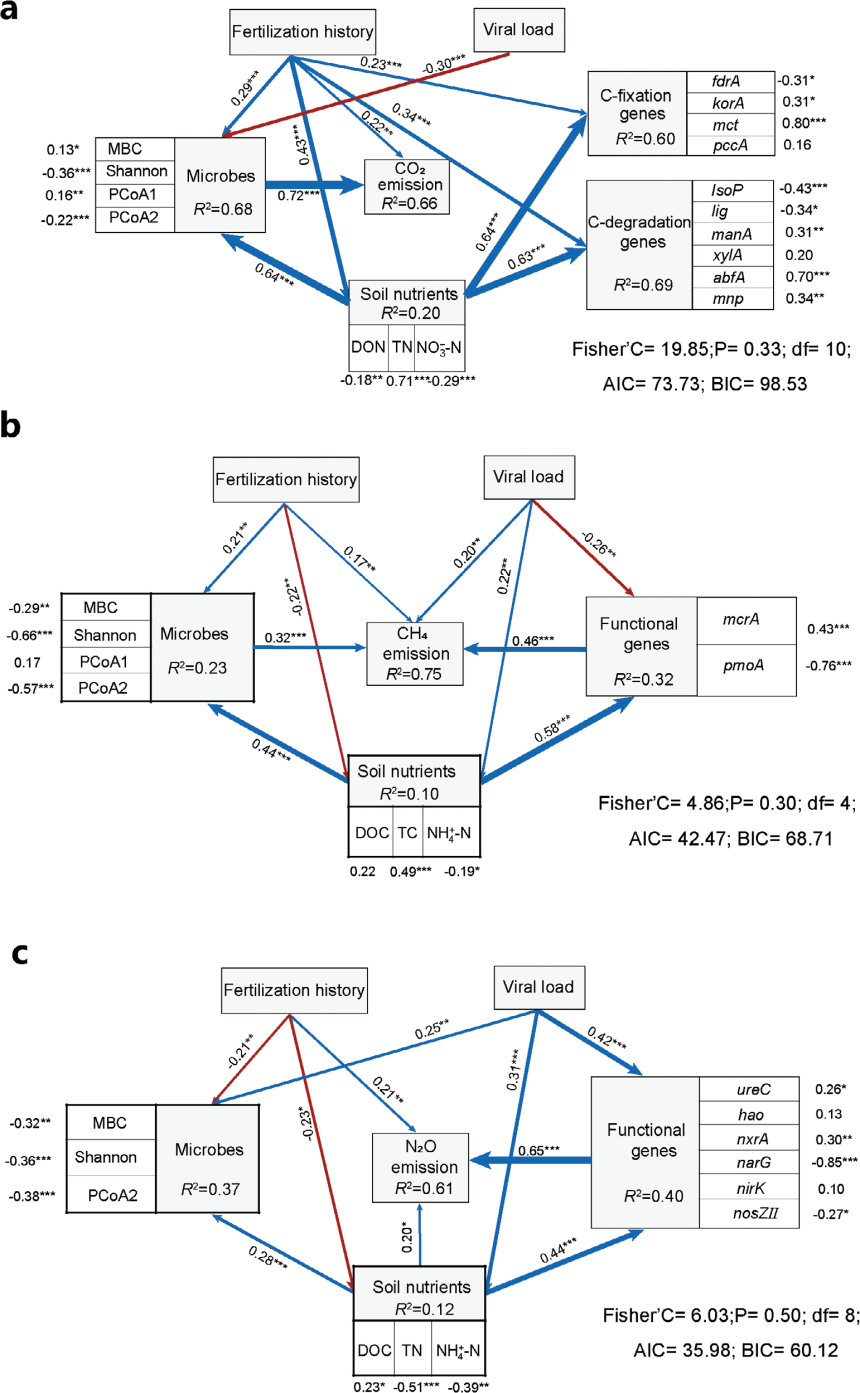
Piecewise structural equation model (SEM) illustrating the direct and indirect effects of fertilization history, viral load, soil nutrients, microbes, and functional genes on a) CO_2_, b) CH_4_, and c) N_2_O emissions. Numbers adjacent to arrows are path coefficients (partial regression) which represent the directly standardized effect size of the relationship. Blue arrows denote positive effects, while red arrows denote negative effects. The fertilization history variable corresponds to the quantities of nitrogen, phosphorus, and potassium in the applied fertilizers. The viral load variable represents the concentration of virus suspension (V0, V1, or V10). Soil nutrients (DOC, DON, TC, TN, NH_4_^+^‐N, and NO_3_^−^‐N), microbes (MBC, richness, Shannon index, and the first two axes of PCoA analysis for microbial community), and functional genes are integrated into the models as composite variables. Numbers adjacent to each box represent the regression coefficient of separate factors on composite variables. Only significant correlations are displayed in the models (^*^ *p* < 0.05, ^**^ *p *< 0.01, ^***^ *p *< 0.001). *R* ^2^ values indicate the proportion of variance explained by all the tested predictors. AIC, Akaike information criterion; BIC, Bayesian information criterion; d.f, degree of freedom.

Using piecewise structural equation modeling (SEM), we tested whether the emissions of each GHG were directly or indirectly associated with changes in soil nutrients, diversity, and composition of the total microbial community as well as changes in the abundance of the functional microbial guilds caused by viral load or soil fertilization history. Our SMEs explained 66.2, 75.1, and 61.8% of the variance found in the CO_2_, CH_4_, and N_2_O emissions, respectively (**Figure** **7**). Soil CO_2_ emissions were indirectly influenced by viral load and soil fertilization history through changes in microbial biomass, diversity, and community composition. Methane and nitrous oxide emissions were also affected by fertilization history and viral load; however, the mechanisms were different from those driving soil respiration. CH_4_ emissions were influenced by the viral load through both direct and indirect effects, including changes in the abundance of methanogens and methanotrophs as well as changes in the total microbial community. In contrast, N_2_O emissions were only indirectly influenced by the viral load through both changes in soil nutrients and in the abundance of N‐cycling microbial guilds.

## 3 Discussion

Although the ecological role of viruses in aquatic ecosystems has been extensively investigated, their impact on soil microbial communities and related functions remains largely unexplored.^[^ ^1^, ^5^, ^19^ ^]^ Additionally, little is known about the consequences of human activities on the influence of viruses in terrestrial ecosystem functioning.^[^ ^13a^ ^]^ Given the important contribution of paddy fields to global biogeochemical cycling, we assessed the impact of soil viruses on microbial communities, soil nutrients, and GHG emissions in paddy soils with three different fertilization histories.

Our results showed major changes in soil microbial biomass and dissolved organic matter after the addition of a high viral load. Previous studies in aquatic ecosystems showed that viruses control nutrient flow by converting host biomass to dissolved organic matter that can be taken up by prokaryotes, diverting microbial biomass away from higher trophic levels.^[^ ^3^, ^20^ ^]^ A key consequence of this “viral shunt” process is a reduction in bacterial abundance accompanied by the increased release of organic matter, which is consistent with our results.^[^ ^20b^ ^]^ Additionally, we noticed that in soils that had a history of fertilization, the addition of native viruses stimulated soil dissolved nitrogen (DON) to a greater extent than non‐native virus addition. One explanation is the presence of a susceptible host reservoir that can be infected directly when native viruses are added; the presence of this reservoir means that the viruses do not need to adapt to new host populations.^[^ ^21^ ^]^ Since the soil C/N ratio is much higher than that in microbial host cells, the release of dissolved nutrients by virus lysis is also expected to have a greater impact on soil nitrogen pools than on carbon pools (Figure 2 and Table S1, Supporting Information).^[^ ^22^ ^]^ These findings provide support for the viral shunt mechanism in soil ecosystems and suggest its dependence on virus sources and soil nutrient levels.

Next, we monitored the emissions of CO_2_, CH_4_, and N_2_O over two months to assess how greenhouse gases were affected by viruses. We found a significant effect of viral load on CO_2_ emissions, with a trend showing higher cumulative emissions after the addition of a low amount of viruses, as previously observed.^[^ ^23^ ^]^ The same trend was observed for the abundances of most carbon fixation and degradation genes (Figure 4a). This may be due to an acceleration of community turnover and/or an enhancement in respiratory metabolism in microbial species after the addition of a low viral load.^[^ ^7^, ^21^, ^24^ ^]^ However, after the addition of a high viral load, a large decrease in prokaryotic diversity and microbial biomass occurred, which ultimately led to a decline in CO_2_ emissions due to soil respiration. Notably, we found that this effect of the high viral load was mostly detected in the soil H (Figure 3a). This might be related to differences in microbial communities between soils with varying fertilization histories. Thus, structural equation modeling highlighted the importance of diversity and composition of the prokaryotic community in the effect of virus addition on CO_2_ emissions (Figure 7). In line with our results, soil respiration is described as a broad function that is widely distributed and performed by a wide range of organisms, and therefore is likely to be affected by any shift in microbial diversity and abundance.^[^ ^25^ ^]^

Previous works showed that organic fertilization could have either a positive or negative impact on CH_4_ emissions, with such an effect also depending on the sampling time.^[^ ^26^ ^]^ Here we found that after harvesting the early rice, i.e., 3 months after the addition of organic fertilizers, CH_4_ emissions were lower in soil H compared to the other two soils (Figure 3b,c). This could be due to the stimulation of CH_4_ uptake by certain organic fertilizers.^[^ ^26a^ ^]^ Accordingly, using ^13^CH_4_ labeling, a previous study from the same plot as ours reported the highest anaerobic oxidation of methane (AOM) rate under organic fertilization.^[^ ^27^ ^]^ We also found that high viral load significantly enhanced both CH_4_ and N_2_O emissions in most soils (Figure 3b,c). These two potent GHGs, which result from a narrow range of functions carried out by specialized groups of microorganisms, were mostly indirectly influenced by virus addition through changes in the abundances of the genes encoding key enzymes involved in the production and consumption of these GHGs (Figure 7b,c).^[^ ^28^ ^]^ In particular, changes in CH_4_ emissions were mirrored in the QMEC results, which showed an increase in methanogens (*mcrA)* accompanied to a lower extent by a decrease in methanotrophs (*pmoA)* at high viral load compared to V0 and V1 (Figures 4b and 7b). In line with our findings, experimental evidence from incubation of soil microcosms with ^13^C─CH_4_ indicated that viruses could infect methanotrophs belonging to *Methylocystaceae* and drive carbon flows in microbial food networks.^[^ ^8^ ^]^

Characterization of prokaryotic communities showed that viruses could also profoundly impact the diversity and composition of these communities. This, together with the large decrease in microbial biomass, provides empirical evidence for strong top‐down control imposed by soil viruses (Figure 5a). While the “Kill the Winner (KtW)” hypothesis posits that dominant prokaryotic populations are more likely to be infected by viruses, enabling minority populations to increase in frequency and maintain microbial diversity, we found a decrease in both richness and Shannon diversity in soils after the addition of a high viral load (Figure 5).^[^ ^21^ ^]^ It is important to note that lysogenic viruses cannot be specifically separated using tangential flow filtration, which limits our ability to distinguish lytic and lysogenic viruses in the present study. Mitomycin C or fluorescent labeling techniques could be employed in the future to investigate the distribution characteristics of lysogenic viruses under varying virus‐to‐host ratios.^[^ ^29^ ^]^

Identification of ASVs that were significantly affected by virus addition revealed that most of the ASVs belonging to the *Desulfarculaceae*, *Desulfocapsaceae*, and *Syntrophobacteraceae* families within the phylum *Desulfobacterota*, as well as to the *Desulfotomaculales* and *Desulfitobacteriaceae* families within the phylum *Firmicutes* were detrimentally impacted at a high viral load (Figure 6 and Tables S9–S11, Supporting Information). Interestingly, prokaryotes affiliated with these families have been described as having a dissimilatory sulfur metabolism. Together with previous studies identifying widespread virus auxiliary metabolism associated with sulfur, our results suggest that viruses may have a strong impact on sulfur cycling, which should be explicitly addressed in the future.^[^ ^10c^ ^]^ In contrast, we also observed significant increases in the relative abundance of several ASVs upon virus addition (Figure 6 and Tables S9–S11, Supporting Information). Since we used inactivated virus at the highest load as a control (V0), this positive effect was not due to the input of elements present in virus particles that could facilitate prokaryotic growth, such as carbon, nitrogen, or phosphorous.^[^ ^19a^ ^]^ Instead, the release of organic matter from microbial cells during viral lysis may have benefited the remaining copiotrophic taxa, such as the phylum Proteobacteria, which is consistent with the “viral shunt” hypothesis. This is supported by the greater impact of a high viral load on microbial community composition in soil L and M than in soil H (Figure 5e,f), with nutrient input by organic and mineral fertilization lessening the benefit of organic matter released by viral lysis. Accordingly, recent research showed that it was more difficult to detect viral shunts in mesotrophic or eutrophic systems than in oligotrophic systems due to the abundant allochthonous and autochthonous organic input sources.^[^ ^20b^ ^]^ Although we cannot elucidate the mechanisms underlying the observed increase in the relative abundance of several ASVs, possibilities include the fitness benefits conferred by integrated prophages to host cells.^[^ ^30^ ^]^ Alternatively, the killing of competitive strains that are susceptible to virus infection could have caused an increase in the relative fitness of the ASVs that were previously impaired by the inactivated strains. By carrying out the targeted removal of various bacterial taxa, researchers have recently demonstrated the importance of competitive interactions in soil bacterial community assembly.^[^ ^31^ ^]^ Interestingly, several ASVs stimulated by the addition of viruses belong to symbiotic taxa, such as the phylum *Nanoarchaea* and the *Symbiobacteriales* family within *Firmicutes* (Tables S9–S11, Supporting Information).^[^ ^32^ ^]^ More work is therefore needed to understand the cascading effects of viruses on interactions between microbes.

In conclusion, our data provide experimental evidence of the impact of viruses on soil microbial communities and biogeochemical cycles. We showed that viral infection can affect soil microbial community biomass, diversity, and composition, which has consequences for biogeochemical cycling and greenhouse gas emissions. In particular, virus addition showed contrasting effects on CO_2_, N_2_O, and CH_4_ emissions and affected a large fraction of the dominant prokaryotic ASVs in soil. Furthermore, our findings underscore the importance of soil fertilization history in modulating the responses of soil microbial communities and biogeochemical processes to viral infection. The decrease in the relative abundance of many ASVs across diverse prokaryotic families that are known to contribute to sulfur cycling emphasizes the need for future studies that incorporate other nutrients when assessing the role of viruses in terrestrial biogeochemical cycling.

## 4 Experimental Section

### Site Description and Soil Sampling

The soil was collected in July 2020 from a rice field located at the Changsha Research Station for Agricultural and Environmental Monitoring (113° 19′ 52″ E, 28° 33′ 04″ N) in the subtropical region of China. This area had a subtropical monsoon climate with mean annual rainfall and temperature of 1553.7 mm and 16.8 °C, respectively. The experimental field site was established in 1986 using a blocked split‐plot design with double cropping: early rice grown from late April to mid‐July and late rice grown from mid‐July to late October. Three fertilizer treatments were applied with four replicates consisting of soil without fertilizer (L, control), soil with NPK fertilizer (M, 142.5 kg N ha^−1^, 23.6 kg P ha^−1^, and 52.3 kg K_2_O ha^−1^ were applied in late April and 158 kg N ha^−1^, 18.6 kg P ha^−1^, and 67.2 kg K ha^−1^ in mid‐July), and soil with 70% NPK + 30% chicken manure (H, 70% of NPK treatment and 8 t chicken manure h^−1^ applied in late April). Soil samples were collected in July after harvesting the early rice. The physical and chemical properties of the soils are presented in Table S1 (Supporting Information).

The soil was sampled from the experimental site using a stainless‐steel drill. Composite samples of five soil cores were collected from the plough layer of soil (0–20 cm) from each block for each fertilization history. Soil samples were sieved (2 mm pore size) to remove roots, debris, and stones and divided into two parts: one was stored at −80 °C for molecular analysis, and the other was stored at 4 °C before subsequent procedures.

### Preparation of the Virus Suspensions from Soil

Approximately 10 kg of each soil sample was suspended in 10 L of SM buffer (68 mM NaCl, 10 mm MgSO4, 10 mm Tris‐Cl pH 7.5) according to previously described methods, shaken vigorously for 15 min, and centrifuged at 3500 × g for 15 min. The supernatants were then collected and vacuum filtered through 5‐, 3‐, and 1‐µm polycarbonate membranes to remove soil particles.^[^ ^33^ ^]^ Tangential flow filtration (TFF) was used to separate microbes and obtain concentrated virus suspensions.^[^ ^34^ ^]^ Two filters (Labscale® Sampler polysulfone provided by Millipore) with different pore sizes: 0.2 µm and 100 kDa were installed in parallel with a peristaltic pump (BT100‐2J, Longer) running at constant speed (10 rpm) to filter the supernatants. The filters were sanitized according to the manufacturer's instructions using 0.1 m NaOH solution before each sample filtration. The TFF method resulted in 130 mL of concentrated viral suspension (V10). This concentrated viral suspension (V10) was either added directly to the soil, autoclaved to obtain the inactivated virus control suspension (V0), or diluted 10 times with the autoclaved viral solutions to obtain the low‐concentration virus suspension (V1) (Figure S7, Supporting Information).

### Virus Sequencing and Analysis

The concentrated viral suspensions (V10) were used to extract the viral DNA from each soil. To eliminate free DNA from cellular fragments, the virus concentrates were treated with DNase I (Sangon Biotech, China) at 37 °C for 60 min. Universal primers 515F/806R were used to confirm the absence of bacterial DNA contamination in the viral concentrates. Viral DNA was extracted with TaKaRa MiniBEST Viral DNA Extraction Kit. For Illumina sequencing, viral sequence libraries were generated using ALFA‐SEQ DNA Library Prep Kit (EINDROP, Guangzhou) following the manufacturer's recommendations. The prepared DNA library was sequenced on an Illumina Novaseq 6000 platform with 150 bp paired‐end reads mode. The viromic data was submitted to the China National Center for Bioinformation, National Genomics Data Center (CNCB‐NGDC) with accession number CRA018401.

Raw sequencing reads for viromic data (n = 12) was subjected to quality filtering using the BBduk.sh script (https://jgi.doe.gov/data‐and‐tools/bbtools/bb‐tools‐user‐guide/bbduk‐guide/). The filtered reads were separately assembled using metaSPAdes (version 3.15.5) with the “metaviral” mode.^[^ ^35^ ^]^ Only scaffolds with a minimum length of 5000 base pairs (bp) were retained for further analysis. A combination of VirSorter2, checkV, and VIBRANT was used to detect viral contigs from viromic assemblies.^[^ ^36^ ^]^ To generate a non‐redundant viral reference database, all 15884 viral scaffolds were dereplicated at 98% gANI over 85% of the viral genomes using dRep dereplicate and resulting in a total of 5851 virus subspecies.^[^ ^37^ ^]^ Finally, the geNomad v1.3.3 pipeline (genomad end‐to‐end) was used for the taxonomic assignment of viral genomes.^[^ ^38^ ^]^

### Microcosm Setup

The three concentrations of virus suspensions (V0, V1, and V10) obtained by TFF from each soil L, M, and H (namely, LV, MV, and HV) were inoculated into microcosms containing 30 g of soil (L, M, or H) using a reciprocal transplant design. The V1 viral load inoculated in each 30 g microcosm corresponded to the amount of virus extracted from the same amount of soil, while the V10 viral load corresponded to that extracted from 10 times the amount of soil. Overall, the experimental design consisted in three soils with different fertilization histories (L, M, and H) × three virus sources (LV, MV, and HV) × three viral loads (V0, V1, and V10) × four replicates, giving a total of 108 microcosms (Figure 1). All microcosms were incubated at room temperature under sterile conditions for 8 weeks after virus inoculation. The moisture content was maintained at 80% of the water holding capacity via the regular addition of sterile water every 5 days. The images of the transmission electron microscope (TEM) and epifluorescence microscope (EFM) confirmed the presence of diverse viruses at different concentrations in the V1 and V10 suspensions (Figures S1 and S2, Supporting Information).

### Soil Nutrient and Gas Measurements

Dissolved soil organic carbon (DOC) and nitrogen (DON) were measured in 0.5 m K_2_SO_4_ extracts (using a soil‐to‐solution ratio of 1: 7.5 w–v) on a Vario Max CNS analyzer (Elementar Instruments, Mt. Laurel, NJ) after filtering the solutions through 0.45 µm ash‐free cellulose filters (Whatman, GE Healthcare Life Sciences).^[^ ^39^ ^]^ Additionally, soils were chloroform‐fumigated for 24 h to estimate microbial biomass carbon (MBC), MBC was calculated as MBC = E_C_/k_EC_, where *E*_C_ = (organic C extracted from fumigated soils)−(organic C extracted from nonfumigated soils) and *k*_EC_ = 0.45.

The soil total carbon (TC) and total nitrogen (TN) were determined by dry combustion in a Vario Max CNS analyzer (Elementar Instruments, Mt. Laurel, NJ). Nitrate (NO_3_^−^‐N) and ammonium (NH_4_^+^‐N) were extracted with 1 m KCl and determined using a flow injection analyzer (SAN++, Skalar, Holland). All measurements were carried out in triplicate.

The soil gas fluxes of carbon dioxide (CO_2_), methane (CH_4_), and nitrous oxide (N_2_O) were monitored in each microcosm before soil sampling. Gas samples (5 ml) were collected regularly during the 8‐week incubation period (days 1, 3, 7, 14, 21, 28, 35, 42, 49, and 56) in 15 mL gas‐tight syringes at 0 and 40 min after sealing the microcosm. Gas samples were analyzed with a gas chromatograph (Shimadzu GC‐2010 Plus, Japan) equipped with a flame ionization detector (FID) and an electron capture detector (ECD) (Agilent Technologies, Wilmington, DE, USA).

### Soil DNA Extraction and 16S rRNA Gene Sequencing

For each microcosm, total DNA was extracted from 0.5 g of soil using the FastDNA SPIN Kit for Soil (MP Biomedicals, LLC, Solon, OH, USA) according to the manufacturer's instructions. The prokaryotic 16S rRNA gene V4 region was amplified using primers 515F (GTGCCAGCMGCCGCGGTAA) and 806R (GGACTACHVGGGTWTCTAAT). Sequencing was performed on a MiSeq platform with a paired‐end protocol (Illumina, 2 × 250 bp). Demultiplexed paired‐end sequencing reads were processed with the QIIME 2 pipeline.^[^ ^40^ ^]^ DADA2 was used for denoising, filtering, merging, and chimera removal from these sequences and to generate amplicon sequence variants (ASVs).^[^ ^41^ ^]^ Taxonomic annotation of ASVs was performed using the naive Bayesian classifier against the SILVA SSU 138.1 database.^[^ ^42^ ^]^

### Quantitative Microbial Element Cycling (QMEC)

Functional genes related to carbon and nitrogen cycling were quantified by a high‐throughput quantitative‐PCR‐based chip (QMEC) as previously described.^[^ ^43^ ^]^ Amplification was conducted in a 100 µl reaction on a Wafergen Smart Chip Real‐time PCR system (Wafergen, Fremont, CA). All qPCRs were conducted in triplicate for each primer set, and a nontemplate negative control was included in each run. The melting process was automatically generated and analyzed using Smart Chip qPCR Software. Multiple melting peaks or those with amplification efficiencies beyond the range (0.9–1.1) were discarded. A threshold cycle (Ct) of 31 was used as the detection limit.

### Statistical Analysis

All statistical analyses were performed in R version 4.2.1 unless otherwise stated.^[^ ^44^ ^]^ Differences in soil nutrient content, functional genes, microbial diversity, and gas flux between treatments were tested using analysis of variance (ANOVA) with a full factorial design followed by Tukey's post hoc test (*p‐*value < 0.05). False Discovery Rate (FDR) control was applied to address the potential risk of Type I error from multiple comparisons.^[^ ^45^ ^]^ Additionally, Multivariate Analysis of Variance (MANOVA) was conducted on each dependent variable to robustly model the interactions between the independent factors. Normality and homogeneity of the residual distribution were verified, and log transformations were performed when necessary. Model assumptions of normality and homoscedasticity were checked on the model residuals, and variables were transformed when needed to meet model assumptions.

Sequencing data were analyzed with the “*phyloseq*” package.^[^ ^46^ ^]^ After quality control and normalization, 10,776,764 sequences were obtained, and 8,707 ASVs were recovered based on 100% similarity of sequences. Microbial α‐diversity (richness and Shannon index) was calculated. Community compositional change was measured using weighted UniFrac distance. The bar plots representing these values and the statistical tests were performed using Tukey's test. The effect of factors was tested using a permutational multivariate analysis of variance (PERMANOVA).

Differential abundance analysis was performed using the “*DESeq2*” package in R to identify ASVs that were significantly affected, with Benjamini‒Hochberg adjusted *p* values (BH‐adjusted *p* value < 0.01).^[^ ^47^ ^]^ To prevent bias due to low‐prevalence taxa, rarefied data were filtered to remove sequences present in less than 40% of samples. Prokaryotic ASVs exhibiting significant changes were used to build pruned trees using Fasttree software and visualized in iTOL.^[^ ^48^ ^]^

Piecewise structural equation modeling was used to assess the direct and indirect relationships between experimental design (fertilizer history and viral load), microbial community (richness, Shannon index, the first two axes of PCoA analysis for microbial community, and MBC), soil variables (DOC, DON, NH_4_^+^‐N, NO_3_^−^‐N, TC, and TN), functional genes (a total of 38 genes involved in carbon and nitrogen cycling), and three ecosystem processes (CO_2_, CH_4_, and N_2_O emissions). A feature of SEM is that it can be used to delineate the possible effects of one variable on another and then estimate the strength of these multiple effects.^[^ ^49^ ^]^ To reduce the complexity of the model, all measured variables included in the models were first divided into “composite variables” and then included in SEM. The use of composite variables could collapse the effects of multiple conceptually related variables into a single composite effect but not alter the underlying SEM, which helps with the interpretation of model results.^[^ ^49^, ^50^ ^]^ These analyses were conducted using the “*piecewiseSEM*”, “nlme”, and “lme4” packages.^[^ ^51^ ^]^ Fisher's C‐test was employed to assess the goodness of the modeling results, and adjustments were made to the models according to variable significance and the goodness of fit of the model.^[^ ^51^ ^]^

## Conflict of Interest

The authors declare no conflict of interest.

## Supporting information



Supporting Information

Supplemental Table 1

## Data Availability

The data that support the findings of this study are available from the corresponding author upon reasonable request.
