# Supplementary material for: Impact of Viruses on Prokaryotic Communities and Greenhouse Gas Emissions in Agricultural Soils
Source: Adv Sci (Weinh). 2024 Oct 7;11(48):2407223. doi: 10.1002/advs.202407223 (PMC11672255; doi:10.1002/advs.202407223)
Supplement: Supplementary file 1 — Supporting Information [file ADVS-11-2407223-s001.docx]

Supplementary Information for

**Impact of viruses on prokaryotic communities and greenhouse gas emissions in agricultural soils**

Xing Huang^1^, Lucas P. P. Braga^2^, Chenxiao Ding^1^, Bokai Yang^1^, Tida Ge^3,^ Hongjie Di^1^, Yan He^1^, Jianming Xu^1^, Laurent Philippot^4^*, Yong Li^1^*

*Corresponding authors:

Yong Li (liyongcn@zju.edu.cn); Laurent Philippot (laurent.philippot@inrae.fr).

**This file includes:**

Supplementary Methods

Supplementary Figure S1-S7

Legends for Supplementary Tables S1 to S12

**Other Supporting Information for this manuscript includes the following:**

Supplementary Table S1-S12

**Supplementary Methods**

**TEM and EFM examination of virus morphology and abundance**

Viral suspensions were examined with transmission electron microscopy (TEM) to check the integrity of viral particles after the filtration procedure. ^[1]^ For each viral suspension 1 μl was sampled and dropped onto a 400-mesh Cu electron microscopy grid that was supported by a carbon-coated Formvar film and incubated at room temperature for 5 min for natural drying. Then, the grids were stained at room temperature for 2 min with phosphotungstic acid negative staining solution (2% w/w) and dried on a filter paper. Grids were examined in a Tecnai G2 20 TWIN TEM operated at 200 kV at a magnification of 25,000–50,000 at the Bio-ultrastructure analysis Lab of Analysis center of Agrobiology and environmental sciences, Zhejiang University.

Viral abundances in the soils were estimated by direct counting of virus-like particles (VLPs) with epifluorescence microscopy (EFM). ^[2]^ One milliliter of the viral suspension was vacuum filtered through a 25-mm diameter filters consisting of a 0.02-μm pore size Whatman Anodisc at a pressure less than 62 kPa for each soil sample. The filters were stained sample side up for 18 min in 100 μl of SYBR GREEN working solution (1:400) in the dark. Subsequently, the dried filters were counted immediately by EFM (Nikon, Melville, USA).

**Supplementary Figure S1-S7**


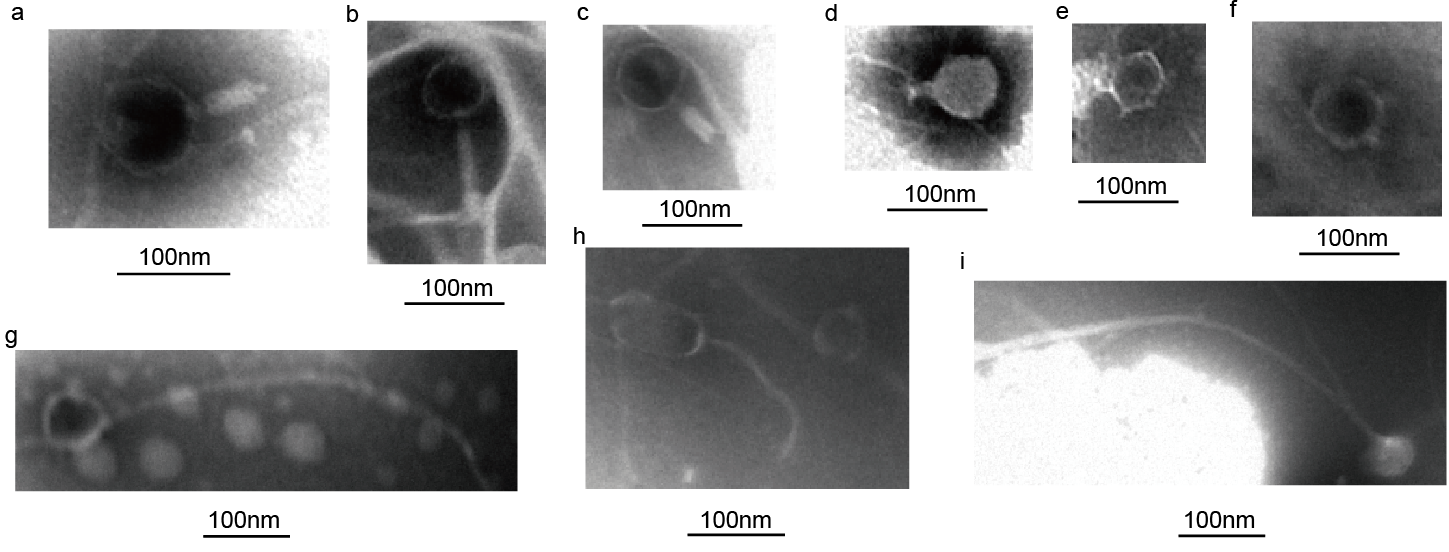


**Fig. S1** Transmission electron microscopic images of viral particles ((**a-c**) Myococcygege, (**d-f**) Podoviridae, and (**g-i**)) Siphoviridae) extracted from soils.


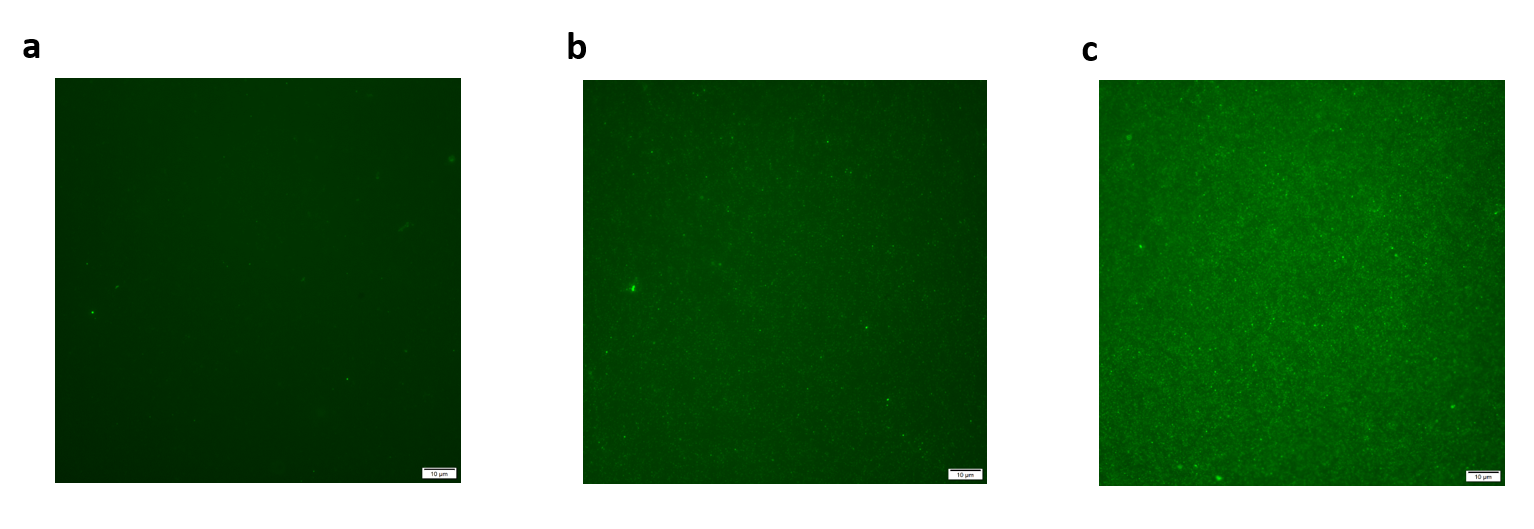


**Fig. S2** Epifluorescence-microscopy image of viral particles (**a**: V0; **b**: V1; **c**: V10) from a soil sample filtered onto a Whatman 0.02 mm Anodisc filter stained with SYBR Gold.


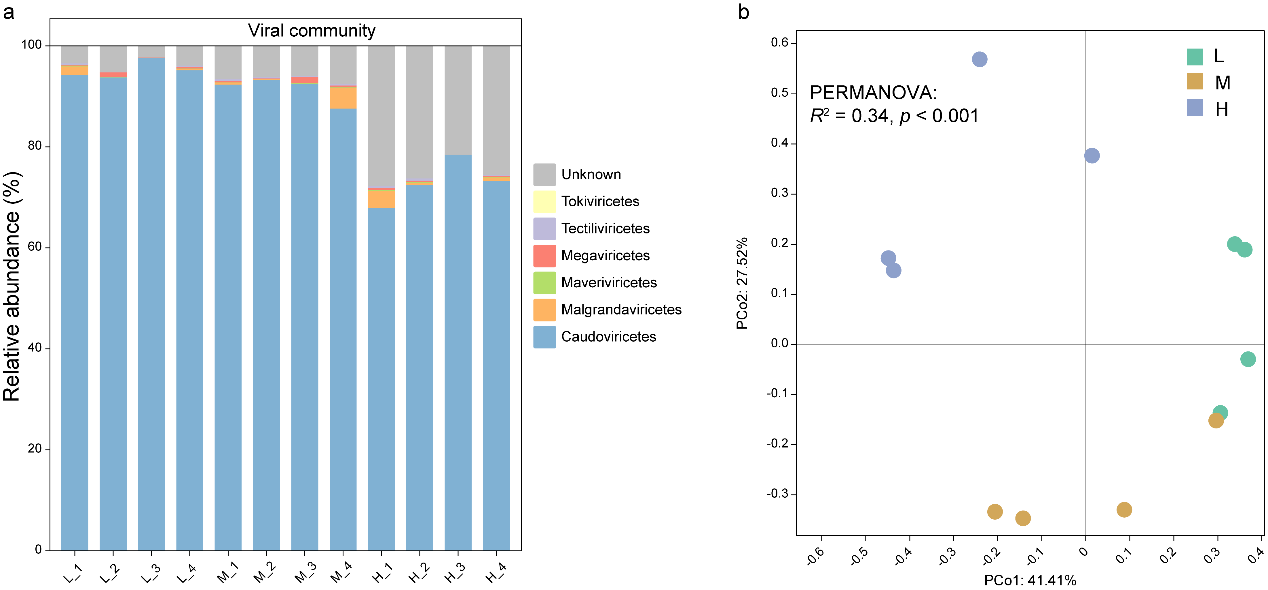


**Fig. S3** Profile of viral communities in virus suspensions obtained from three soil types. The composition of viral (class level) communities from three different locations (**a**). Principal coordinate analysis (PCoA) of viral taxonomic structure colored by sampling sites (**b)**.

**
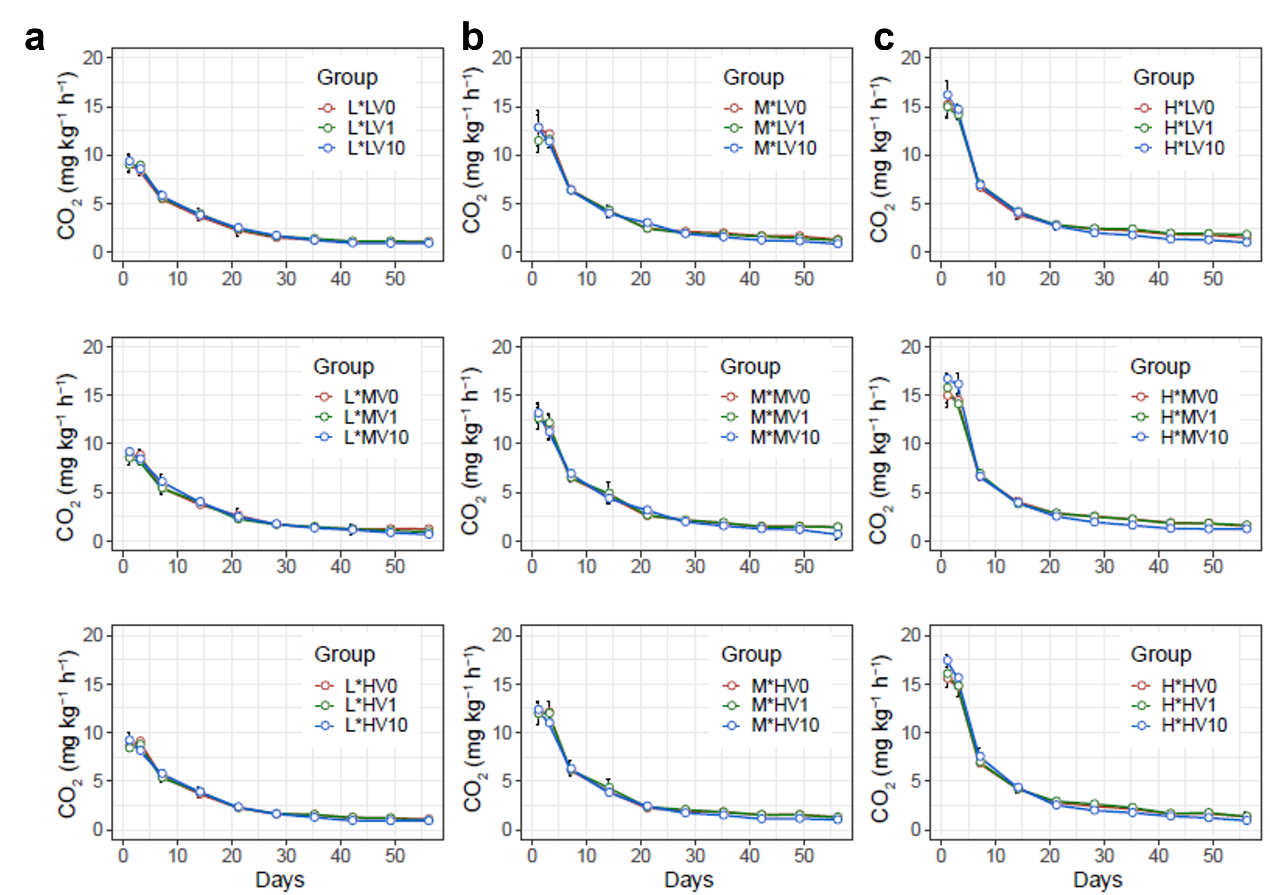
Fig. S4 Dynamics of CO_2_ fluxes.** CO_2_ fluxes from the microcosms containing the soil L **(a)**, M **(b)** and H **(c)**were measured throughout the 8-week incubation period after inoculation with the virus suspensions at different loads (V0, V1, and V10) from the different soils (LV, MV and HV). Rates are presented as the mean of the values measured within each treatment (n=4), with their respective standard deviation.


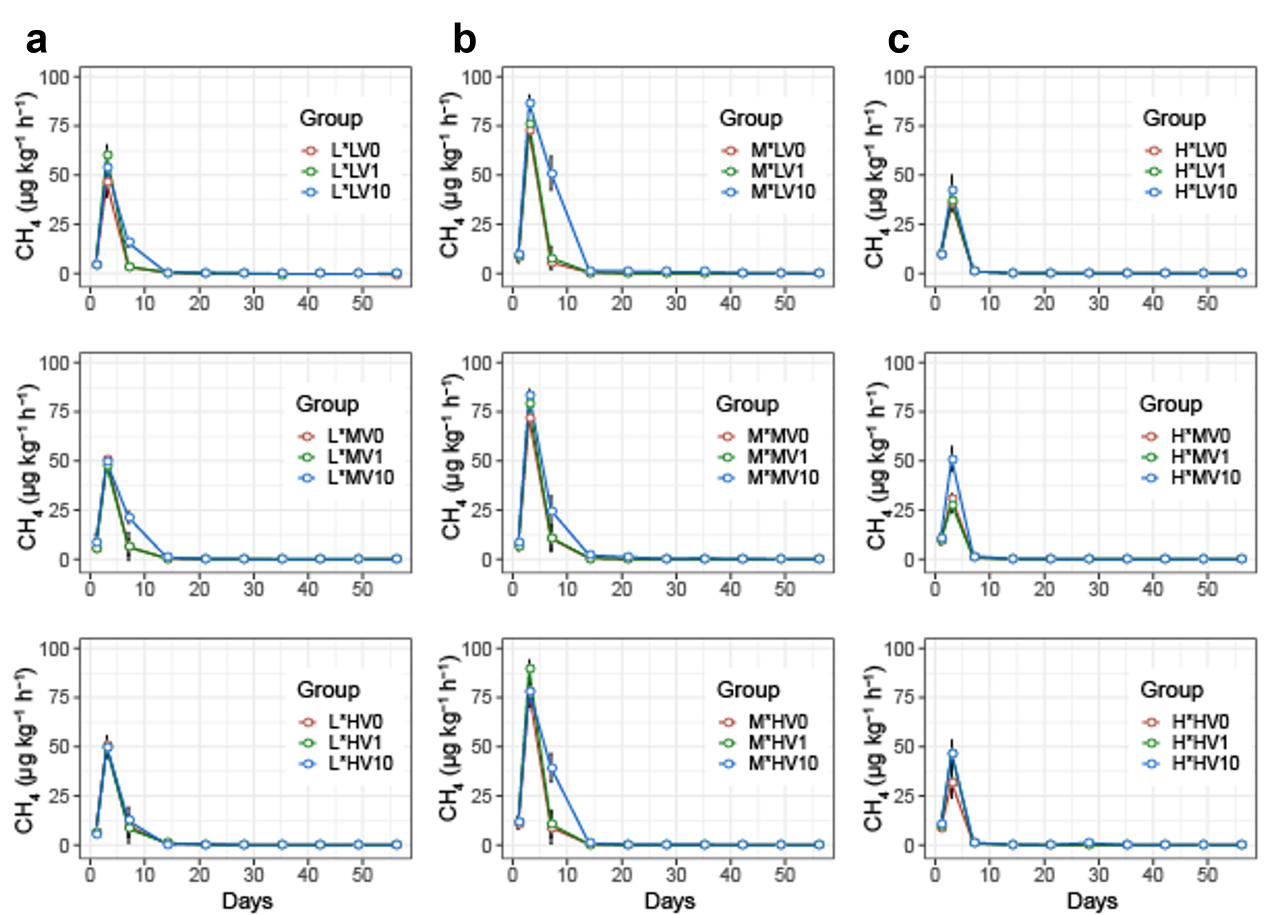


**Fig. S5 Dynamics of CH_4_ fluxes.** CH_4_ fluxes from the microcosms containing the soil L **(a)**, M **(b)** and H **(c)**were measured throughout the 8-week incubation period after inoculation with the virus suspensions at different loads (V0, V1, and V10) from the different soils (LV, MV and HV). Rates are presented as the mean of the values measured within each treatment (n=4), with their respective standard deviation.

**
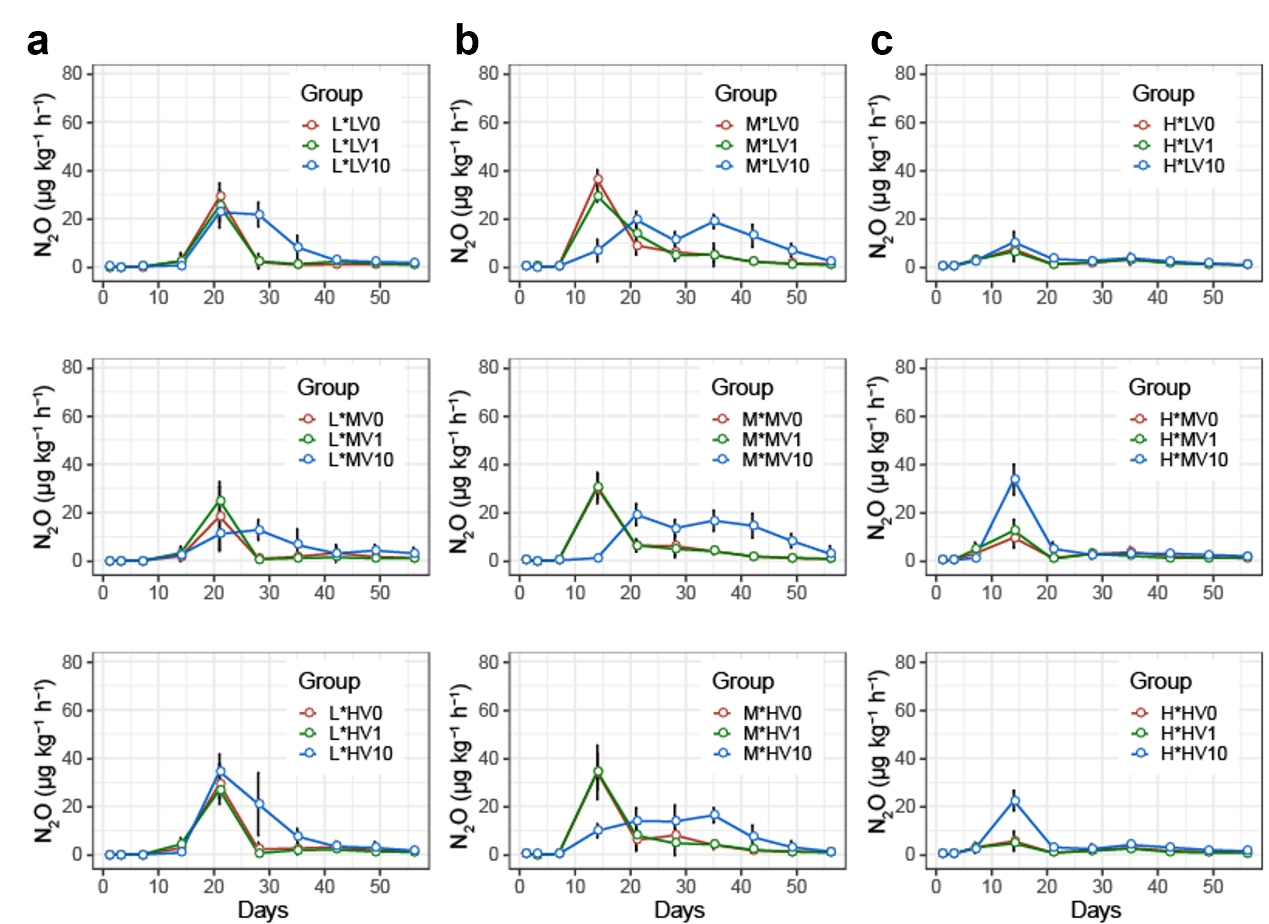
Fig. S6 Dynamics of N_2_O fluxes.** N_2_O fluxes from the microcosms containing the soil L (a), M (b) and H (c)were measured throughout the 8-week incubation period after inoculation with the virus suspensions at different loads (V0, V1, and V10) from the different soils (LV, MV and HV). Rates are presented as the mean of the values measured within each treatment (n=4), with their respective standard deviation.


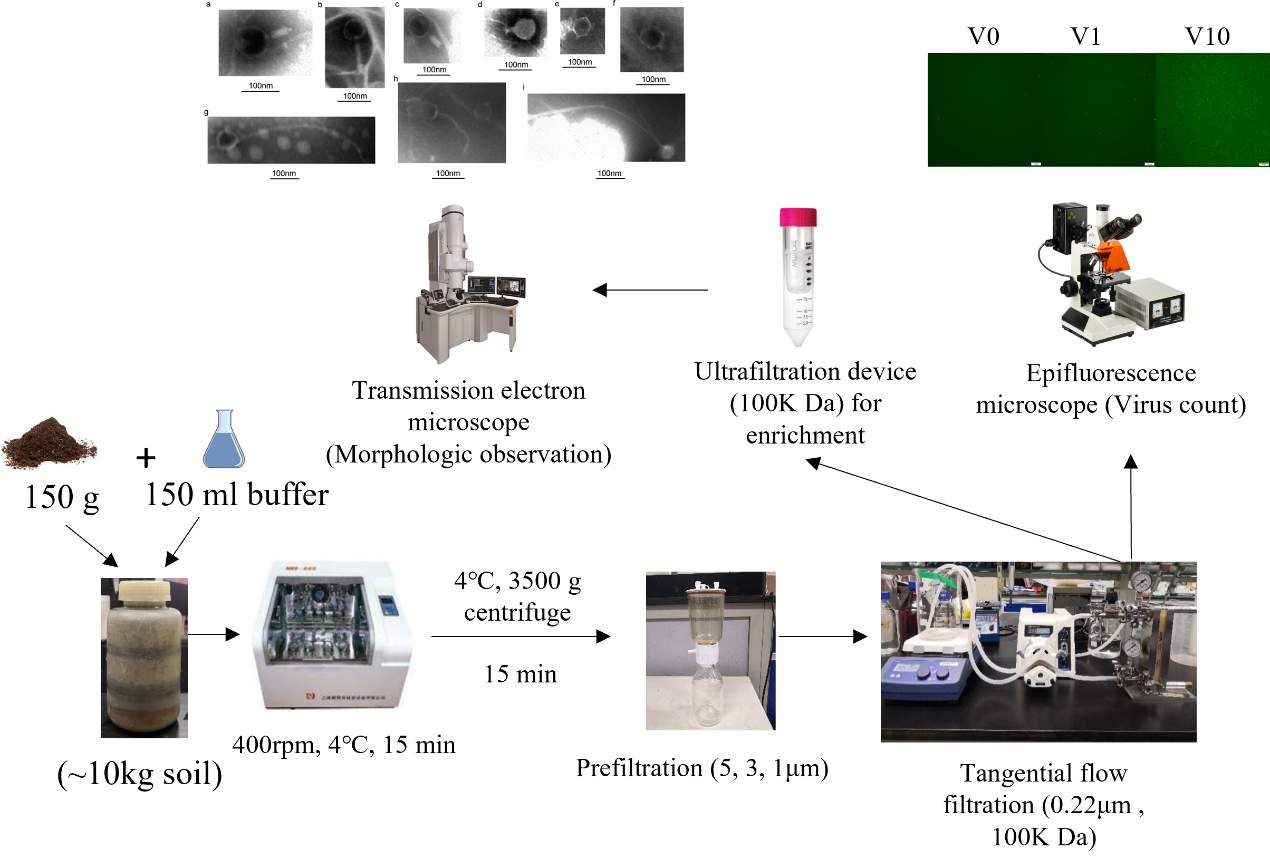


**Fig. S7 Workflow of soil virus isolation and enrichment.** This figure illustrates the experimental procedure employed for the isolation and enrichment of soil viruses in this study.

**Legends for Supplementary Tables S1 to S12**

**Table S1** Description of the soil physicochemical properties among three type of paddy soils. SWC, soil water content; SOM, soil organic matter; DOC, soil dissolved organic carbon; DON, soil dissolved organic nitrogen; TC, total carbon; TN, total nitrogen; NH_4_-N, ammonium nitrogen; NO_3_-N, nitrate nitrogen; AP, soil available phosphorous. The letter in parenthesis indicates significant differences between soil (Tukey’s HSD test).

**Table S2** Three-way analysis of variance (ANOVA) showing the main and interaction effects of virus load (V0, V1 and V10), virus source (native and non-native), and soil fertilizer history (soil L, M and H) on soil nutrients. Significant effect (*p* < 0.05) are indicated in bold. DOC, soil dissolved organic carbon; DON, soil dissolved organic nitrogen; TC, total carbon; TN, total nitrogen; NH_4_-N, ammonium; NO_3_-N, nitrate.

**Table S3** Three-way analysis of variance (ANOVA) showing the main and interaction effects of virus load (V0, V1 and V10), virus source (native and non-native), and soil fertilizer history (soil L, M and H) on greenhouse gas flux. Significant impacts (*p* < 0.05) are indicated in bold.

**Table S4** Three-way analysis of variance (ANOVA) showing the main and interaction effects for soil fertilizer history (soil L, M and H), virus source (native and non-native) and virus load (V0, V1 and V10) on C fixation related functional genes, with degrees of freedom (Df), *F* statistics, and *p* values.

**Table S5** Three-way analysis of variance (ANOVA) showing the main and interaction effects for soil fertilizer history (soil L, M and H), virus source (native and non-native) and virus load (V0, V1 and V10) on C hydrolysis related functional genes, with degrees of freedom (Df), *F* statistics, and *p* values.

**Table S6** Three-way analysis of variance (ANOVA) showing main and interaction effects for soil fertilizer history (soil L, M and H), virus source (native and non-native) and virus load (V0, V1 and V10) on methane metabolism related functional genes, with degrees of freedom (Df), *F* statistics, and *p* values.

**Table S7** Three-way analysis of variance (ANOVA) showing main and interaction effects for soil fertilizer history (soil L, M and H), virus source (native and non-native) and virus load (V0, V1 and V10) on nitrogen cycling related functional genes, with degrees of freedom (Df), *F* statistics, and *p* values.

**Table S8** Three-way analysis of variance (ANOVA) showing main and interaction effects for soil fertilizer history (soil L, M and H), virus source (native and non-native) and virus load (V0, V1 and V10) on microbial biomass carbon (MBC), richness, and Shannon index of microbial community, with degrees of freedom (Df), *F* statistics, and *p* values.

**Table S9** Summed numbers of the ASVs enriched in high or low virus load treatments. The relative abundances of changed ASVs in initial soil are shown in parentheses.

**Table S10** The ASVs enriched in high or low virus load treatments at soil L. Log_2_ Fold Change values were shown to indicate difference between low and high viral loads.

**Table S11** The ASVs enriched in high or low virus load treatments at soil M. Log_2_ Fold Change values were shown to indicate difference between low and high viral loads.

**Table S12** The ASVs enriched in high or low virus load treatments at soil H. Log_2_ Fold Change values were shown to indicate difference between low and high viral loads.

**References:**

[1] K. Wommack, R. Hill, M. Kessel, E. Russek-Cohen, R. Colwell, *Appl. Environ. Microbiol.* **1992**, *58* (9), 2965.

[2] a) G. Sun, J. Xiao, H. Wang, C. Gong, Y. Pan, S. Yan, Y. Wang, *MethodsX* **2014**, *1*, 197; b) A. C. Ortmann, C. A. Suttle, in **2009**, Ch. Volume 1: Isolation, Characterization, and Interactions.
